# The Role Played by Public Universities in Mitigating the Coronavirus Catastrophe in Brazil: Solidarity, Research and Support to Local Governments Facing the Health Crisis

**DOI:** 10.3389/fsoc.2021.610297

**Published:** 2021-02-11

**Authors:** Cristiano Alencar Arrais, Graciella Corcioli, Gabriel da Silva Medina

**Affiliations:** ^1^ Faculdade de História da Universidade Federal de Goiás, Goiânia, Brazil; ^2^ Setor de Desenvolvimento Rural, Escola de Agronomia da Universidade Federal de Goiás, Goiânia, Brazil; ^3^ Faculdade de Agronomia e Medicina Veterinária, Universidade de Brasília, Brasília, Brazil

**Keywords:** health crisis, research, outreach, solidarity, federal universities, impact of COVID-19

## Abstract

This study aims to assess the impacts of the Covid-19 pandemic in Brazil and how it has been dealt with by both the government and in civil society. To this end, we examine the Brazilian public health system and the measures taken by the Bolsonaro Government that led to Brazil being ranked second in overall Covid-19 infections in the world through August 2020. In the absence of national leadership facing the Covid-19 health crisis, we list a set of science-based initiatives promoted by Brazilian public universities in connection with local governments, NGOs and communities as a means of mitigating the consequences and spread of the pandemic. This study is based on the consultation of institutional material published by universities summarizing their research and outreach initiatives. Results reveal that university initiatives included: 1) Alerts to society on the risks of the pandemic, with an emphasis on establishing observatories that assisted local governments and civil society in understanding the evolution of the disease, as well as in implementing measures for its prevention; 2) Direct assistance to local communities, with emphases on the addition of beds in university hospitals for treating patients with Covid-19 and on the manufacturing of personal protective equipment and; 3) Research to find solutions to prevent and treat the disease, with emphases on the development of tests for Covid-19, as well as on carrying out phase 3 vaccine trials. Through these measures, Brazilian public Federal Universities played a key role in supporting both civil society and local governments in mitigating the impacts of the pandemic.

## Introduction

The social crisis caused by the Covid-19 pandemic has posed a major challenge to world leaders, whose models of response to the health crisis have resulted, in some cases, in the strengthening and cohesion of national politics and, in others, in the political weakening of heads of state and a deepening crisis of political legitimacy. The medical protocols established thus far may still need to be adjusted due to the very recent nature of the pandemic. The European model for addressing the health crisis has been the large-scale adoption of lockdowns, but when applied to Latin American countries, this has had questionable results (Blofield et al., 2020; Gardini, 2020; Hausmann, 2020) both in terms of its application in territories significantly larger than those of most European nations, and in terms of the adherence within socially heterogeneous populations.

However, some basic strategies to contain the pandemic such as social distancing, hand sanitizing, and wearing protective masks have also become standard procedures in most regions of Latin America. Increasing research has already pointed out that the use of masks for protection, is not restricted to the prevention of spreading the virus by means of an individual mechanical barrier. The adoption of this mechanism for individual protection by the community reinforces both the sense of personal control, protection and the sense of collective responsibility as a symbol of respect within the community combating the pandemic (Cheng et al., 2020; Schünemann et al., 2020; Szczesniak et al., 2020).

In Brazil, the pandemic has had significant human costs. In August 2020, the country had the second largest number of infected people in the world, behind only the United States of America. In addition, as Covid-19 spread, a considerable part of the economically active population has either lost their jobs or have had their salaries decreased through furloughs or wage cuts. Preliminary studies indicate that families with incomes up to two times the minimum wage would be the most impacted, with income losses 20% higher than average Brazilian families (Domingues et al., 2020).

This study aims to examine the impacts of the Covid-19 pandemic in Brazil and the process of confronting this health crisis by the Federal Government and organized civil society. To this end, it examines Brazil’s health system and the impact of the Covid-19 pandemic on Brazilian society. It also examines the relationship between the political environment encouraged by President Jair Messias Bolsonaro and the spread of the virus in the country, which led to Brazil having the second highest number of infections and deaths in the world through the end of August 2020. Finally, we analyze the processes of social mobilization of the national public university network in the country, operating as public agencies with autonomous management, whom have assumed a role in promoting research for the production of vaccines and drugs, as well as in solidarity actions with local populations due to the lack of national leadership and these populations’ social vulnerability while facing this health crisis.

## Materials and Methods

This study was prepared using a socioeconomic dataset produced by the Brazilian Government and both national and international research institutions that allowed reconstruction of the impacts of the epidemiological crisis in Brazil. Brazilian public universities are deemed as a set of autonomous entities interconnected by an interuniversity network that has been established in the last decades as an important political actor, distinct from the State and other traditional political actors. Official data released by the Brazilian public universities from their websites, a compilation of data produced by the National Association of Leaders of Federal Institutions of Higher Learning (ANDIFES) about the activities developed by 68 Brazilian Public Universities, and data provided by the Ministry of Education (MEC) was used in these analyses. Additionally, information from other universities in Latin America was used in order to establish a parameter about the role played by universities in Latin America in mitigating the effects of the pandemic and their relationship with their respective central governments. It was done based on consultation of institutional material published by universities and interviews with professors from the Universidad de Chile, the Universidad Nacional Autónoma de Mexico and the Pontificia Universidad de Perú. The study quantifies the actions developed according to three central indicators: solidarity actions, support to the management of the epidemiological crisis, and research. In addition to the total number of actions carried out by the Federal Education Institutions (IFEs), the two largest universities in each of the five regions of the country were analyzed based on the Web Ranking of Universities 2020. The selection criteria for sampling the studied universities was based on the principle of the Brazilian Constitution stating that “universities enjoy didactic-scientific, administrative and financial and patrimonial management autonomy, and will obey the principle of inseparability between teaching, research and extension” (Brasil, 1988). Due to the suspension of teaching activities in the first phase of the pandemic, research and outreach activities gained prominence. Outreach actions were subdivided in order to distinguish between support activities local managers such as state governors or mayors and activities with a direct impact on society. In the latter case, the different target audiences were characterized regardless the number of people served. This sample aimed at comparing quantitative data with qualitative information about the actions developed by the selected universities in order to examine the impacts of such actions on socially vulnerable populations.

## Results

### The Brazilian Universal Healthcare System and the Evolution of the COVID-19 Pandemic

The first recorded Covid-19 death in Latin America occurred on February 26 in Brazil. Since then, countries have adopted distinct strategies aimed at containing the spread of the virus and mitigating its consequences (Litewka and Heitman, 2020). In Brazil, challenges from the Covid-19 pandemic have been combatted by the Universal Healthcare System (SUS), the national public healthcare network, inspired by the National Health Service (United Kingdom) and instituted by Law 8.080 of September 19, 1990. SUS encompasses primary care, of medium and high complexity, emergency services, hospital care, epidemiological, health and environmental surveillance actions and services, and pharmaceutical assistance. In addition to vaccination programs and home care services, SUS is present throughout the country, and in 2019 seven out of ten Brazilians depended exclusively on SUS for access to healthcare (FIOCRUZ, 2020; IBGE, 2020).

In 2018, Brazil had 452,801 doctors when including public and private networks, which corresponds to the ratio of 2.18 doctors per 1,000 inhabitants. However, this indicator does not consider regional and municipal inequalities, which indicate the vast concentration of these doctors to be in the most favored regions and municipalities. The medical ratio per thousand inhabitants is 2.81 in the Southeast, 2.36 in the Central West, 2.31 in the South, but only 1.41 in the Northeast and 1.16 in the North. Municipal inequalities are even greater when one observes that while some cities, like Vitória, ES (in the Southeast) maintain a ratio of 12 doctors per 1,000 inhabitants, there are other municipalities in the North and Northeast regions that do not even reach 1 doctor per 1,000 inhabitants, maintaining a ratio far below the national average. This is the case in the states of Pará in the North with only 0.97 doctors and Maranhão in the Northeast with only 0.87 doctors per 1,000 inhabitants (Medical Demography, 2018).

A universal healthcare system is important in Brazil because it decreases social inequalities, among other aspects, and SUS becomes the only medical-hospital assistance institution in the most isolated regions or those regions with great socioeconomic inequalities. In 2017, SUS had about 335,000 doctors, 202,000 nurses, and 12,500 dentists in 129,544 public and private healthcare facilities, considering that “even with a public and universal healthcare system, SUS’s performance and expansion were imbricated to the private sector, especially when we consider the contracted services/contracted workers” (Viacava et al., 2018 p. 1753).

The pattern of development of the Covid-19 contagion and mortality curve in Brazil, as well as the curve’s acceleration, have a direct relationship with the universal healthcare system for the treatment and planning to combat this health crisis. When examining the model of action practiced by high-performance healthcare systems such as Japan, Hong Kong and Singapore, Legido-Quigley et al. (2020) highlight three major challenges to be faced in the event of a prolonged health crisis:

The first is that integration of services in the health system and across other sectors amplifies the ability to absorb and adapt to shock. The second is that the spread of fake news and misinformation constitutes a major unresolved challenge. Finally, the trust of patients, health-care professionals, and society as a whole in government is of paramount importance for meeting health crises (Legido-Quigley et al., 2020 p. 849).

Comparatively, SUS performance in Brazil fell short of what was expected in relation to the three key aspects: a) integration with a view to avoiding overloads in the healthcare system, b) managing fake news about the crisis, and c) increasing trust in the governance system.

- Although the first case of coronavirus was reported on December 31, 2019 and the WHO decreed a pandemic on March 11, 2020, integration was found to be disorganized as the healthcare system was unprepared to confront Covid-19 due to the lack of infrastructure, equipment and skilled professionals prepared to face the pandemic. The symptom of this unpreparedness was the rapid collapse of the ICU network (Intensive Care Units) in public and private hospitals, leading to the construction of Covid campaign hospitals throughout the country. However, through the month May, when the contagion and mortality curves had reached their highest point, only 47% of the Covid campaign hospitals were in operation. President Jair Bolsonaro’s strategy of political confrontation and his insistence on rejecting internationally established sanitary protocols reinforced his alienation from many governors and mayors. A judicial battle took place over the definition of legal authority to establish rules of isolation, quarantine, and restriction of transportation and traffic on highways when local and state governments were faced with a federal attempt to impose rules contrary to those of horizontal isolation and social distancing. The decision was finally taken to the highest court in the country, the Federal Supreme Court (STF). Unanimously, the supreme court justices reaffirmed the competence of states and municipalities to decide, without exempting the Federal Government’s responsibility, in addition to their legitimacy to define which activities should be suspended during the Coronavirus pandemic. The result was the lack of a unified protocol of actions between municipalities, states and the Federal Government regarding the contingency of the epidemiological crisis, along with the adoption of local measures that were sometimes divergent and/or overlapping. This dispute between the various government spheres also affected the process of sharing and distributing public resources for combating the pandemic. According to the National Health Council-SUS deliberative collegiate instance-the Ministry of Health (MS) has R$ 39.0 billion in resources to combat the new Coronavirus, but 66% of the budget is frozen. Of the R$ 11.4 billion earmarked for the purchase of respirators, masks and other PPE items needed for the population, health workers and to equip health units, only 25% of resources were released by the MS. The transfers of federal resources to states and municipalities reached only 41% and 44% of the total available, respectively (CNS, 2020).
- Transparency in the dissemination of information to the population has been considered paramount in controlling the pandemic, but unfortunately this did not occur in Brazil. Since the first Covid-19 cases in Brazil, the President of the Republic has regularly published misleading, controversial or unsubstantiated news about the spread of the pandemic, to the point where independent fact-checkers on Instagram and Twitter have labeled posts he has made as *fake news*. The Ministry of Health has also avoided transparency. There have been three different ministers during the pandemic, one of them an interim minister with no formal training in healthcare. At the beginning of June 2020, after suppressing data regarding the number of contagions and deaths from the Ministry of Health’s website, the official accounting of the number of contagions and deaths from Covid-19 was altered, in order to make it more difficult to obtain and advertise the data. Furthermore, the President of the Republic is a fervent supporter of the use of hydroxychloroquine in the treatment of Covid-19. He has promoted its use on his personal social media accounts, in interviews, and live videos, as the preferred drug of choice for the treatment of Covid-19. After removing the two Health Ministers during the pandemic, Bolsonaro finally succeeded in establishing a governmental protocol for the use of the drug to treat the epidemic according to his criteria: “Pazuello [interim Health Minister] decided to change the orientation and wrote that in any situation, to prescribe chloroquine,’ so that the doctor could have his freedom” (Bolsonaro, 2020).
- As the government has been unstable since the beginning of President Jair Bolsonaro’s administration, trust in governance has been difficult to come by. In an opinion poll conducted by the Datafolha Institute on August 11^th^ and 12^th^, 88% of survey respondents did not even know the name of the current Minister of Health, 49% believed that the Federal Government had not done what was necessary to avoid the growing number of Covid-19 deaths, and 33% of respondents considered President Jair Bolsonaro very responsible for the advancement of coronavirus in Brazil (16% considered him a little responsible and 49% did not consider him responsible) (DATAFOLHA, 2020). The Brazilian situation is different from that of nations whose leaders played a key role in the responsible handling of the pandemic, such as New Zealand. In countries where the federal government has adopted a serious stance in relation to the pandemic, citizen trust in their leader of state, as well as in the information provided by the health authorities, has been much greater and this has helped in citizens adhering to government measures of social isolation and individual protection.

### The Brazilian Federal Government’s Approach to the Covid-19 Pandemic

Contrary to the suggested global strategy of pandemic control, the President of the Republic rarely appears in public with an individual protection mask, despite the guidelines of the Ministry of Health itself and individual state laws that establish the mandatory use of this PPE. These and other public attitudes, on the part of the Executive Branch, are part of a strategy of political confrontation based on a social agenda of an ultraconservative nature, adopted since the presidential campaign of 2018 and sustained throughout his election in the same year. For over two decades Brazil has been a federative republican democracy and after the election of four previous presidents post-military dictatorship, Bolsonaro’s government’s priorities include re-establishing the facility for citizens’ right to bear arms and ammunition, establishing military schools for elementary and secondary education, the relaxation of environmental protections, the relaxation of protections for the territories of traditional populations (indigenous, quilombolas, and river peoples), and a favoritism toward governmental privilege of dialogue with high-ranking military and conservative religious leaders to the detriment of organized civil rights movements (specifically targeting the LGBT community, the black and minority movements, labor unions, the landless rural workers movement, and indigenous tribes, among others).

Social networks have been fundamental instruments for the dissemination of false and misleading information and hate messages against individuals and institutions that do not fit this particular vision of the President of the Republic. Public Federal Universities-a network of higher learning with 69 institutions distributed throughout Brazil-were one of the main targets of these institutional attacks. Former Minister of Education Abraham Weintraub, removed from office after a public threat to STF justices, became known for comparing university professors to “fat zebras” and for calling higher learning institutions “centers of communist indoctrination.” This campaign, aimed at destroying the public image of the Federal Institutes of Higher Learning (IFEs), was accompanied by a sharp reduction in the budget allocated to education, especially higher education, whose contingency rate was already 30% in 2019. By 2021, the budget reduction is expected to reach 18.2% (G1, 2020a).

This has led to the negative perception of the Presidency of the Republic which has had consequences on the actions taken by the Federal Government to combat the effects of the pandemic. Since the first Provisional Measures aimed at combating the pandemic were established on March 13, through the end of June, a heterogeneous and sometimes conflicting set of federal regulations has been released, edited and re-edited. Some of them are in direct conflict with the guidelines of both the World Health Organization (WHO) and the first Brazilian Ministry of Health itself, such as the inclusion of re-opening businesses such as fitness centers, beauty salons and barbershops in the category of “essential services” (Decree 10.344 of May 11, 2020), the mass production of hydroxychloroquine by army laboratories, and alterations in the dissemination of national Covid-19 case and mortality data. This type of erratic management of the Covid-19 crisis has resulted in a higher mortality rate in comparison to the majority of other countries in North and South America, second only to the United States of America.

**FIGURE 1:**
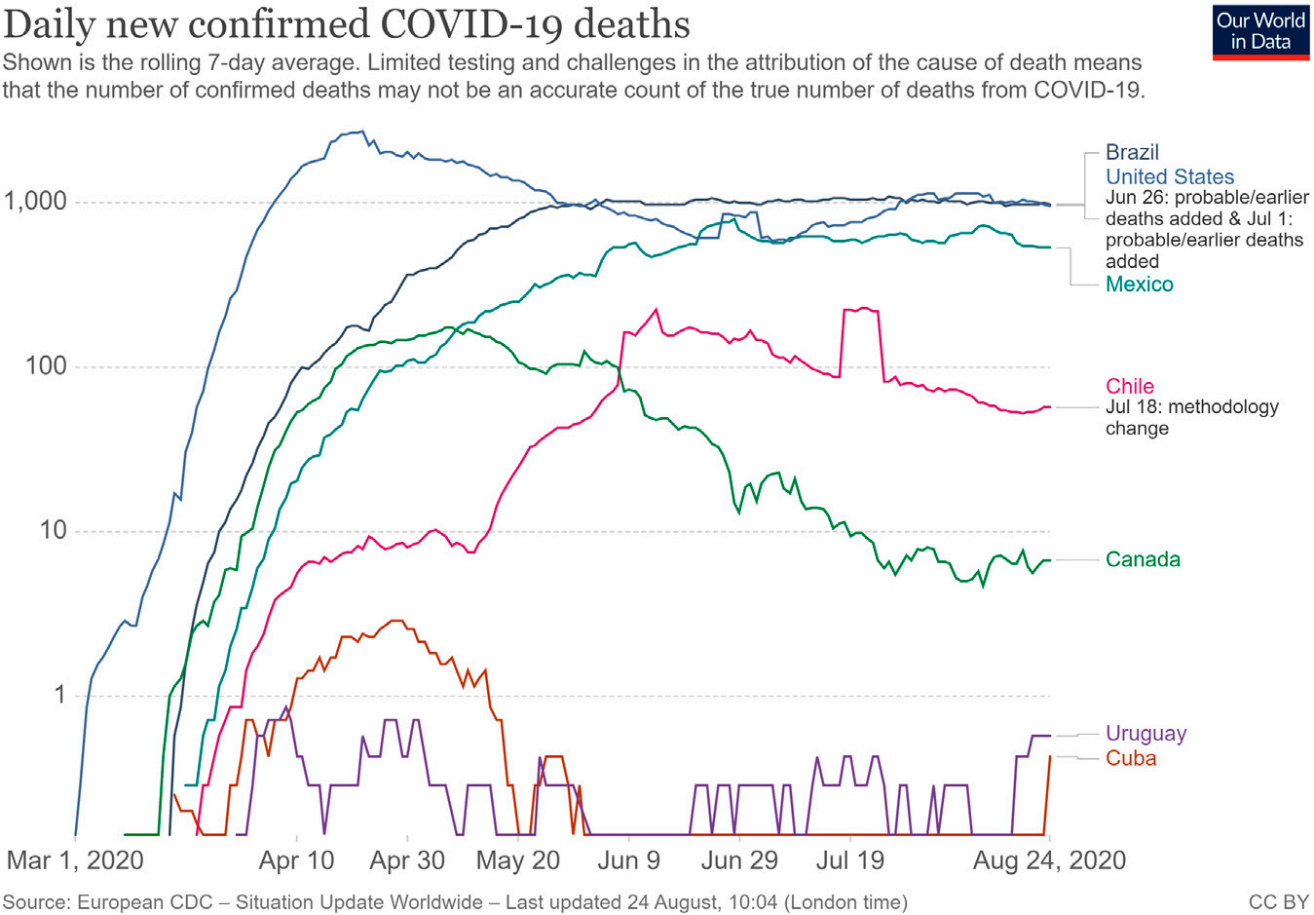
Comparison between the trajectory of mortality numbers confirmed by COVID-19 in the countries of the combined North and South American continent. Source: Elaborated by the authors from the data obtained on OurWorldInData.org.

As it is possible to see in Figure 1, when comparing eight countries with different HDI levels (Canada: 0.922; United States: 0.920; Chile: 0.847, Uruguay: 0.808; Cuba: 0.778; Mexico: 0.767 and Brazil: 0.761) (UNDP, 2019), the continuous weekly mortality rate in Brazil remained at a level close to 1,000 daily deaths for a longer period, from the third week of May until the end of August 2020. With the exception of the United States, which shows a fluctuating trajectory in the number of recorded deaths, all of the other countries reached their mortality peaks and then indicated declines in mortality. In contrast to observations in most European and Asian countries, where the mortality curve peaked, and was followed by a gradual drop and then smaller waves, the Brazilian case is different. In Brazil, these indicators maintained a consistency for months which transformed the “first wave” of deaths into a prolonged plateau that only showed discrete signs of decline at the end of August.

Thus, while the average levels of daily deaths caused by Covid-19 seem to have reached a maximum, their maintenance over months indicates that basic control measures have had their efficiency compromised given a low adherence of local populations. Several studies associated population’s adherence to measures of social distancing with the political orientation of local leaders (Allcott et al., 2020; Barrios and Hochberg, 2020; Painter and Qiu, 2020).

Economically, the government delayed the adoption of measures to protect domestic business markets, causing inertia in production activities. This delay is partially explained by the difficulty of the economic team in overcoming the liberal orthodoxy of the portfolio holder, Economic Minister Paulo Guedes. It took over two months to implement the first measures from the declaration of a pandemic situation by the WHO on March 11 until the first application of government financial resources (Table 1).

**TABLE 1:** Economic measures of the Brazilian government to combat the socioeconomic effects collateral effects of the Covid-19 pandemic through July 2020.

Provisional measure	Objective	Total value (x billions [BRL])	Value applied to the target public until through Julye 2020 (x billions of reals [BRL]) and consequences in the internal market
MP 937	Emergency aid for vulnerable people	R$ 254.0	R$ 167.62. The aid pledged by the government of R$ 200.00 was raised to R$ 600.00 by the national congress. Until the beginning of June, more than 11 million requests for aid remained "under analysis".
MP 929	Expansion of family aid^1^	R$ 3.04	R$ 0.37. Through ordinance 13.474, the federal government tried to transfer part of this package to the secretary of communication of the president of the republic, responsible for official advertising campaigns.
MP 936	Emergency benefits of employment and salary maintenance	R$ 51.64	R$ 18.24. The objective was to provide the means for the maintenance of company and job activities, making proportional reductions in the work day and wages or the temporary suspension of work contracts for up to 90 days. Workers, in this case, would have continued access to unemployment insurance or job benefits proportional to their wage reduction and the company would be mandated to not fire employees for the same period as the benefit. With uncertainty of the pandemic's end, there was little adherence to this measure.
MP 939	State and municipal compensation for the loss of resources from the participation fund	R$ 76.19	R$ 39.94. Such limited resources committed to this plan are associated with pressure from the federal government to end social distancing measures and restrictions on economic activities. Loss of revenue in states and municipalities has fostered criticism of the actions local public managers have taken during the pandemic especially from local businessmen.
MP 943	Grant for payroll financing	R$ 34.0	R$ 3.91. In theory, a measure that would benefit medium and large businesses with public financing for the payroll of employees. This measure was delayed in passing on resources from the BNDES to credit agencies, in addition to finding bureaucratic barriers in private financial institutions and high interest rates that made the measure practically unviable.
MP 977	Quotas for operation and credit guarantee funds	R$ 35.90	R$ 20.90. Credit line directed at medium and large businesses for investment. Despite a greater necessity during the pandemic, small and medium enterprises did not have access to the credit line due to the rigid demand for fiduciary guarantees from banking institutions. A support program for micro and small entrepreneurs outlined later in May.

^1^ Social program created in 2003 and instituted by Law n° 10.836 of January 9, 2004, focused on improving the income inequality, with conditions, in the area of education and health. By 2016, it assisted over 13.8 million poor families in Brazil, especially those categorized as black and brown.

The result of the failure to adopt immediate and effective economic measures was the loss of 1,092,578 jobs in the first half of 2020 (a difference of −18.5% in relation to the same period of 2019), a fall of 11.4% in GDP in the second quarter of 2020 when compared to the same period of 2019, and the projection of a further decline in GDP of 5.52% (CAGED, 2020). Currently, Brazil has almost 50% of the economically active population, unemployed (IPEA, 2020).

### Federal Universities Face the Covid-19 Pandemic

In different Latin American countries, universities have joined efforts with governments in order to mitigate the effects of the pandemic. The consultation of institutional material published by universities and interviews with professors revealed a close collaboration between universities and central governments. In Peru, the Pontifical University (PUCP) dedicated itself to the manufacture of mechanical respirators to assist the country's hospital structure, with the President of the Republic visiting the facilities and declaring the relevance of the services provided by the institution. In Mexico, twenty-eight hospitals and university laboratories comprised the network of entities authorized by the Institute of Diagnosis and Epidemiological Reference (InDRE), directly linked to the Presidency of the Republic, to carry out the diagnosis of Covid 19 throughout the country. In Argentina, the partnership established between universities and the central government is symptomatic of the spirit of cooperation between State and society. President Fernandes provided financial support to the actions taken to combat the pandemic, especially in the metropolitan region of Buenos Aires, the largest urban center in Argentina. In addition, the central government is funding research projects in the area of social and human sciences through the “*PISAC-COVID-19: La sociedad argentina en la postpandemia”* program (DW, 2020; Hernandez, 2020; Kulemeyer, 2020; Lossio, 2020; UNAM, 2020).

These experiences are an indicator of the importance of new political actors in the process of strengthening civil society in Latin America, a region historically dominated by populism and military dictatorships (Moore, 1983). Cooperation between civil society and the government is a relatively recent phenomenon in Latin America, which is typical of the redemocratization process that took place after military dictatorships and aim the autonomy of politically organized groups in relation to the State. At the same time, scholars in Latin America agree that although the mechanisms of participation and empowerment of organized civil society have shown strong evolution in recent decades, they are still fragile. (Smulovitz and Peruzzotti, 2000; Eckstein and Wickham-Crowley, 2003).

Since the reestablishment of democracy in the late 1980s, following the phenomenon that occurred throughout Latin America, the Brazilian model of higher education has undergone profound changes and is currently characterized by a trend toward accessibility and inclusivity (Torres and Schugurensky, 2002; Arocena and Sutz, 2005). In addition to this, Article 207 of the 1988 Federal Constitution established “didactic-scientific, administrative and financial management autonomy, and [institutions] will obey the principle of inseparability between teaching, research and extension,” while its deans are appointed by the President of the Republic from a triplicated list established by the university community. This infraconstitutional regulation ensured that the network of federal universities, although belonging to the state’s bureaucratic structure, have certain autonomy regardless of the political orientation of ruling governments.

Public policies aimed at the inclusion of social groups historically discriminated against, such as indigenous peoples, African Americans and students from public schools have increased the scope and size of the university education system. In 2001, the federal public education system had 67 institutions, which had jumped to 110 institutions by 2018. In spite of the significant growth in the number of Federal Institutions (IFs), this number only represents 4.34% of the entire Brazilian higher learning system and 15.68% of educational enrollments. This is because private higher learning has also been encouraged along with a strong increase in public funding for them through scholarships and student funding. As a result, the private sector represents 88.21% of all higher learning institutions and 75.41% of educational enrollments in Brazil (INEP, 2019).

As far as Brazilian scientific production is concerned, the proportion of participation of public/private universities is inverted. The country ranks 13th in terms of global scientific production and more than 95% of the Brazilian scientific production made available on an international basis comes from public universities, with emphases on areas such as agriculture, medicine and health (Clarivate Analytics, 2019). Studies focused only on the analysis of scientific articles indexed in international databases (Web of Science, Scopus and others) reached a similar conclusion, also noting the increase in scientific production in recent decades for the non-traditional educational regions of Brazil (Diniz-Filho et al., 2016; Souza et al., 2016). Surprisingly, the socioeconomic crisis caused by the Covid-19 pandemic has led to a trial by fire for Brazilian public universities. University hospitals, laboratories and research groups have made substantial efforts and instituted large-scale programs in short timeframes along with the assistance of local mayors and state governors to fight the pandemic with a great deal of success.

The ANDIFES report (National Association of Leaders of Federal Institutions of Higher Learning) highlights the actions of 68 IFEs, the participants of the research in the fight against the Covid-19 pandemic. Table 2 shows the intense effort made by Brazilian Federal Universities to provide direct care to local populations, with an emphasis on increasing ICUs for the treatment of people contaminated with Covid-19. More than 3,000 hospital beds were allocated throughout the entire university network, with some institutions contributing over 800 beds alone. Even universities that did not allocate beds for Covid-19, such as UFAM, received regular patients in their facilities in order to make more beds in public hospitals available for Covid-19 patients. It is also worth mentioning that over 1,000 research initiatives were developed in the universities related to Covid-19 during this time, and the almost 500 voluntary solidarity actions were carried out by professors, employees and students from the universities to give assistance to local populations. In addition to actions aimed at the external community, there are also actions of solidarity within the university community, from the suspension of in school face-to-face activities, student socioeconomic surveys to plan for the return of classes, guaranteeing the constitutional principle of inclusiveness, and adding learning support measures (curricular restructuring for asynchronous classes when possible) for remote classes as the new semester in August 2020 began for most institutions.

**TABLE 2:** Quantity of actions developed by Brazilian Federal Universities organized by type.

Action^1^	National total^2^	Federal public universities^3^									
		UFRJ	UFRGS	UFMG	UFSC	UNB	UFBA	UFC	UFG	UFPA	UFAM
Beds in university hospitals^4^	3,158	164	93	894	75	35	80	17	38	58	0
Research studies	1,260	15	50	111	129	56	4	17	12	9	13
Production of hand sanitizer	113	3	2	2	3	2	2	4	1	1	0
Production of PPEs	121	3	2	3	4	1	3	5	1	1	7
Covid 19 testing	71	2	1	9	1	10	1	0	1	1	0
Educational campaigns	1,226	1	11	17	20	41	10	75	43	5	7
Solidarity actions	482	6	19	80	12	19	13	8	35	1	17
Municipal government partnerships	255	2	7	2	4	1	1	2	5	1	0
State government partnerships	112	3	4	5	1	1	3	1	1	1	1
Other relevant actions	1,343	16	19	37	16	32	3	71	27	4	24
Human development index^5^	0.761	0.761	0.746	0.731	0.774	0.824	0.660	0.682	0.735	0.646	0.674

^1^ Principal actions disseminated on the sites of the universities sampled.

^2^ IFEs Emergency Actions in the fight against the new coronavirus. Report prepared by ANDIFES and sent to the authors by Cogecom/ANDIFES (2020).

^3^ Principal Brazilian Federal Universities distributed among the five regions of the country, according to the Web Ranking of Universities available at: http://www.webometrics.info/en/Latin_America/Brazil. Accessed on July 22, 2020.

^4^ The total number of beds includes beds owned by the University Hospitals and beds made available through partnerships for the construction and operation of hospitals for the Covid campaign.

^5^ Human Development Index of Brazil (PNUD, 2019) and the states that comprise the Federative Republic (Censo, 2010).

In Table 2, one can see that the number of actions are significantly higher for universities located in the most economically developed regions of the country, as opposed to regions where smaller social indicators are found in the consolidated data. The Southeast and South of Brazil contain the best internationally ranked universities. However, in spite of these differences, the significance of the actions carried out by each of these institutions can only be measured if we consider the type of social impact caused within the context of their region. Thus, in a region with low socioeconomic indicators containing traditional communities such as river peoples, indigenous tribes and quilombolas such as the North and Northeast regions, charity and solidarity actions can have a more important direct impact than research actions.

As observed in Table 2, there was a concentrated effort on three different contingency fronts in the fight against the harmful effects of the Covid-19 pandemic:

1. Alert society to the risks of the pandemic, with an emphasis on creating observatories that would help local governments and civil society to understand the evolution of the disease, as well as implementing measures for its prevention. In addition, the participation of university specialists in the process of planning and building contingency plans implemented by state and municipal public managers is observed, which has subsidized decision-making both in relation to strategies for social distancing, suspension of non-essential production activities, and in the process of gradually reopening economic activities;
2. Provide direct service to the local community, with an emphasis on increasing beds in university hospital ICUs for the treatment of patients with Covid-19 and the manufacturing of personal protection equipment (PPEs). Since Brazil has a population around 206 million, with an average HDI of 0.761 (UNDP, 2019) - ranging from 0.418 to 0.862 depending on the municipality (Censo, 2010)-and a poor or extremely poor population estimated to be around 31.8% of the total population, actions directly associated with mitigating the effects of the pandemic are just as important as the discovery of a vaccine or more permanent solution. As a result, the most vulnerable populations, such as the indigenous and river peoples’ communities, necessitated solidarity actions implemented by the IFEs;
3. Focus research on finding solutions to prevent and treat Covid-19, with an emphasis on the development of improved tests, as well as cooperation for the development of phase 3 vaccine testing. In the latter case, since the epidemic has disseminated at such a large-scale throughout the country, Brazil has become one of the most opportune environments for mass testing. In addition to this, Brazil is noted for efficient scientific production and a top-class laboratory structure that has allowed for the development of partnerships with some of the most important worldwide research centers on Covid-19.

Table 3 allows us to examine the social impact of the actions developed by Federal Universities within their regional contexts in detail. Solidarity initiatives for direct assistance to the local population were oriented for more vulnerable populations that, although they did not reach a broad public, contribute to the protection and preservation of populations representative of Brazilian cultural diversity.

**TABLE 3:** Sampling of the actions carried out by the Federal Universities in the fight against Covid-19.

University^1^	Actions^2^		
	Programs	Management support	Studies
UFRJ	1. Partnerships with over 30 civil organizations to organize campaigns collecting essential products and organizing primary health care teams in the communities.	1. Plans of action to combat Covid-19 in the communities (slums) of Morro do Alemão, Cidade de Deus, Maré, Rocinha and Santa Marta.	1. Analysis on the impact of the Covid-19 pandemic on the quality of life of women living in areas of social vulnerability in Rio de Janeiro.
	2. Training of primary health care teams in communities.	2. Website development for real-time monitoring of Covid-19 cases at the university hospital.	2. Development of a test to detect antibodies to Covid-19. The test called S-UFRJ costs less than 1.0 US$.
UFRGS	1. Development of business design alternatives for small businesses to ensure the continuity of their activities during the pandemic.	1. Production and donation of 100 thousand facial protectors for health professionals and other essential services.	1. Evaluation of the efficacy and safety in using plants in the ethnopharmacological management of viral respiratory infections, such as Covid-19.
	2. Training of a therapeutic group that provides emotional support to public school teachers.	2. Development of the Coronavis website, which is a tool to support the visual analysis of Coronavirus data.	2. Development of a prototype of a N95 mask decontamination chamber that uses ultraviolet radiation and allows the equipment to be reused.
UFMG	1. Assistance with gestation, delivery and birth of the indigenous Pataxó, Maxakali and Xakriabá groups, with the distribution of Covid-19 educational material.	1. Elaboration of a mathematical model to evaluate the percentage of Covid-19 underreporting of cases in Brazil.	1. Project mapping the mass Covid-19 testing in Betim.
	2. A group of alumni have launched a campaign to collect computer equipment to enable remote teaching of public school students.	2. Development of the CovidLP app that provides short and long term forecasts for Covid-19.	2. Dog and cat testing to assess the risk of Covid-19 transmission between humans and animals.
UFSC	1. Distribution of food purchased from family farmers to populations living in a state of social vulnerability.	1. Detection of Covid-19 particles in sewage samples collected in 2019.	1. Study on the possibility of using the BCG Vaccine to combat Covid-19.
	2. Creation of a support group for people in mourning due to Covid-19.	2. Development of an app for mobile phones that allows detecting and notifying people who have had close contact with suspected or confirmed cases of Covid-19.	2. Development of a low-cost mechanical respirator prototype.
UNB	1. Telehealth Program for virtual care to the indigenous population of the Federal District.	1. Epidemiological monitoring and reporting to the population and the local DF government.	1. Participation in the phase 3 tests for a Covid-19 vaccine with the objective of testing safely at a large scale and with efficacy of the CoronaVac product.
	2. Creation of the ATHOS Project (Technical Assistance for Housing of Social Origin) to reduce the vulnerability of the population living in precarious conditions to contamination by zoonoses.	2. Development of COVID19 Tracker - Application for tracking social interaction in an epidemic scenario.	2. Implementation of a multi-user level 3 biosafety laboratory.
UFBA	1. Distribution of 4,697 masks and 4,586 hygiene kits for the homeless in the city of Salvador.	1. Test standardization for Covid-19 detection with the use of saliva.	1. Participation in the phase 3 tests for a Covid-19 vaccine with the objective of testing safely at a large scale and with efficacy of the CoronaVac product.
	2. Drive Sapeca, which collected food and cleaning supplies for institutions accredited by the Municipal Council for the Rights of Children and Adolescents.	2. GeoCombate group developed a multidisciplinary study that identifies neighborhoods in Salvador that are most vulnerable to Covid-19.	2. Development of sink prototypes to function as community hygiene points in neighborhoods with irregular water supply.
UFC	1. Monitoring the health of the elderly in shelters by the medical residents of the School of Medicine.	1. Development of a low-cost respirator for medical use.	1. In silico studies to evaluate the efficacy of eight molecules (synthetic peptides) that interact with the virus to prevent it from communicating with human protein.
	2. A multidisciplinary team carries out actions to prevent covid-19 for people living on the streets of Fortaleza-CE.	2. Development of the Predictive Monitoring System (SIMOP) that follows the evolution of COVID-19 in Fortaleza-CE.	2. Development and implementation of a portable sink for people on the street.
UFG	1. UFG Solidarity Extension Action to raise funds for food baskets to be donated.	1. Consultancy with the Public Health Emergency Operations Committee of the State of Goiás through mathematical models to forecast advances of the pandemic and plan control actions.	1. Use of Artificial Intelligence (AI) to discover effective drugs to combat Covid-19.
	2. Donation of kits with individual protection equipment, thermometers, oximeters and Covid-19 tests to indigenous communities in Mato Grosso and Tocantins.	2. Implementation of the Tendinha subproject, which aims to test children and adolescents as a means to assess the spread of the disease.	2. Making of cushions for injury prevention during Covid-19 treatment.
UFPA	1. Development and distribution of information booklets about Covid-19 in several indigenous languages.	1. Production of PCR tests to perform Covid-19 identification tests in partnership with the state government.	1. Physiotherapeutic assistance for people recovering/recovered from Covid-19.
	2. Elaboration of an information booklet about the basic care needed during the Covid-19 pandemic.	2. Development of the Data Observatory Project: COVID-19, which provides a webpage with a series of graphics that makes it easier to monitor the development of the disease in the country.	2. Conducting research on the behavior of the elderly in the pandemic.
UFAM	1. Acquisition and distribution of PPEs for rural and river inhabitants and support to the commercialization of agricultural products.	1. A Covid-19 observatory for case monitoring in the state of Amazonas with an alert system for civil and public managers.	1. Computational research on the efficiency of pharmaceutical drugs to combat Covid-19.
	2. Amazon Campaign against Covid-19 to collect food and cleaning products for donation to indigenous families.	2. Definition of the Covid-19 epidemiological curve in Manaus, which revealed to the effectiveness of social distancing for flattening the curve.	2. Identification of patent-free drugs as an alternative treatment for Covid-19.

^1^ Principal Brazilian Federal Universities distributed among the five regions of the country, according to the Web Ranking of Universities available at: http://www.webometrics.info/en/Latin_America/Brazil. Accessed on July 22, 2020.

^2^ Actions disseminated on the websites of the universities sampled: www.ufrj.br; ww.ufrgs.br; www.ufmg.br; www.ufsc; www.unb.br; www.ufba.br; www.ufc.br; www.ufg.br; www.ufpa.br; www.ufam.br.

In regions with a greater representation of indigenous communities, special attention was given since these communities are fragile, often formed by only a few dozen individuals, and face possible extinction. In Brazil, the urban indigenous population live mostly in municipalities with high risk for COVID-19 and 22% of the rural indigenous population live in municipalities with high epidemiological risk (FIOCRUZ, 2020a). The total indigenous population is less than 900,000 people in 304 distinct groups, some have relative integration with non-Indigenous peoples, such as the Yanomani, while others are isolated individuals who refuse external contact, such as the Kawahiva. However, 31,469 cases of Covid-19 and 797 deaths were still registered through September 12, 2020 (APIB, 2020), representing a lethality rate of 2.53% in these groups.

In cities with inadequate housing, such as slums (favelas), specific actions were adopted taking social vulnerability and the difficulty of enforcing social distance measures into account. Data from 2015 show that “about 11.4 million people live in precarious territories, several of which are made up of slums. About 12.2% of the population under theses precarious conditions (1.4 million) are in Rio de Janeiro. These slums are characterized by difficulties in access, high density of buildings, precarious housing and insufficient supply of essential public services, such as water supply and garbage collection.” Additionally, in these areas there is high incidence of diseases such as tuberculosis, hypertension, heart disease, diabetes and high homicide rates (FIOCRUZ, 2020b).

As an example of these actions, UFPA and UFPI (UFPA, 2020; UFPI, 2020) prepared pamphlets on the prevention of spreading Covid-19 in indigenous languages. While UFRJ has developed tailored action plans for five specific communities (favelas) in Rio de Janeiro to combat Covid-19.

According to data from the Ministry of Education, these actions by Federal Institutions of Education aimed at mitigating the impacts of Covid-19 had impacted approximately 30 million people, which represents 14% of the country’s population by the end of July 2020 (MEC, 2020). These numbers are even more remarkable when compared to Tables 2, 3, since in 2018, statistics show that 13.5 million Brazilians live below the poverty line (G1, 2020b).

## Discussion

The evolution of the Covid-19 pandemic in Brazil has highlighted at least two opposing social and political trends:

Firstly, the failure of the Executive Branch to lead and manage the Covid-19 pandemic, leading to an ever rising mortality rate from the pandemic’s inception through August 2020. President Jair Bolsonaro’s null approach has contributed to the Brazilian healthcare system’s (SUS) difficulties to cope with the pandemic, fractured the relationship between science, the people and policies, and discredited internationally recognized scientific evidence, all factors that could have potentially mitigated the effects of the pandemic. As a consequence, in August 2020, Brazil remained second in the number of Covid-19 deaths, behind only the United States, and all while facing an unprecedented economic crisis.

Secondly, in the midst of the President’s null approach to the pandemic, the scientific effort made by Federal Universities did play an important role in mitigating the effects of the pandemic. Although Federal Universities are public entities, the autonomous character of their management system assured by the Federal Constitution of 1988, enabled the necessary independence from the ruling central government. As public entities with management autonomy, together with other scientific institutions, these universities helped alert society about the risk of the pandemic, provided direct assistance to local communities, and developed research for solutions to both prevent and treat the disease. Thus, while organically linked to the Ministry of Education, but enjoying fundamental administrative and didactic-scientific autonomy, Brazilian Federal Universities have self-organized and established close collaborations with municipal and state decision-makers aimed at developing possible solutions to be implemented by public institutions during the health crisis.

The effort made by these institutions can be measured by the volume of actions and strategies developed within the public Federal University system, by the influence these actions had on modeling pandemic contingency strategies in municipalities and states, and through the strengthening of the relationship between science and the people as a mechanism capable of fighting fake news and misinformation about the spread and prevention of Covid-19.

Since 2019, there has been strong campaign to discredit the network of Federal Universities by the Bolsonaro’s government and defund the areas of education and health through budgetary constraints. The Covid-19 pandemic has magnified the intense battle between these public institutions designed to produce scientific and social applied knowledge for the betterment of Brazil and the Brazilian ruling President. Through financial squabbling, the improper nomination of non-elected deans, and public smear campaigns, the ruling government intends to weaken the universities and everything they represent (science, the autonomy of thought, and respect for diversity and inclusivity), all while in ideological reverence to the extreme conservative social base that elected him in 2018 (comprised of representatives of agribusiness, weapons manufacturers, the military, and Christian evangelicals). Thus, the President intends to remain politically viable for the next presidential election.

On the other hand, the actions developed by universities had a significant impact in mitigating the effects of the pandemic in Brazil and the various projects focused on combating the spread of COVID-19 in socially vulnerable communities have been just as vital as the research projects for the development of vaccines and therapies. Despite current political threats to the existence of Brazilian public Federal Universities, the volume of their actions, the diversity of populations they assisted and the products they delivered to Brazilian society indicate they are resilient and socially engaged.

## Data Availability

Publicly available datasets were analyzed in this study. This data can be found here: APIB, (2020). ARTICULAÇÃO DOS POVOS INDÍGENAS. http://emergenciaindigena.apib.info/dados_covid19/. Accessed September 12, 2020 CAGED (2020). Ministério do Trabalho e Emprego. Programa de Disseminação de estatísticas do trabalho. http://pdet.mte.gov.br/images/Novo_CAGED/Jul2020/2-apresentacao.pdf. Accessed August 24, 2020. CNS (2020). Conselho Nacional de Saúde. Boletim Cofin. Ministério da Saúde. http://www.susconecta.org.br/wp-content/uploads/2020/06/Boletim-2020_0617_T1_ate_15_RB-FF-CO_resumida-1.pdf. Accessed August 26, 2020. Demographic Censo, 2010 (2010). Índice de Desenvolvimento Humano dos Estados. IBGE, 2010. https://www.ibge.gov.br/cidades-e-estados. Accessed September 11, 2020. IBGE (2020). Pesquisa Nacional de Saúde (PNS). https://www.ibge.gov.br/estatisticas/sociais/saude/9160-pesquisa-nacional-de-saude.html?=&t=resultados. Accessed September 12, 2020. INEP (2019). Instituto Nacional de Estudos e Pesquisas Educacionais Anísio Teixeira. Sinopse Estatística da Educação Superior 2018. Brasília: INEP, 2019. http://inep.gov.br/web/guest/sinopses-estatisticas-da-educacao-superior. Accessed June 16, 2020. IPEA (2020). Instituto de Pesquisas Econômicas Aplicadas. Carta de conjuntura – Mercado de trabalho. Nº 37, segundo trimestre de 2020. https://www.ipea.gov.br/portal/images/stories/PDFs/cc47_nt_pnad.pdf. Accessed September 11, 2020. MEC (2020). Ministério da Educação. Coronavírus: monitoramento nas instituições de ensino. http://portal.mec.gov.br/coronavirus/. Accessed August 14, 2020.
